# Evaluation of the Microstructure and Mechanical Properties of the Butt-Welded Joints of Spiral Pipes Made of L485ME (X70) Steel

**DOI:** 10.3390/ma16196557

**Published:** 2023-10-05

**Authors:** Lechosław Tuz

**Affiliations:** Faculty of Materials Engineering and Industrial Computer Science, AGH University, 30-059 Kraków, Poland; ltuz@agh.edu.pl

**Keywords:** natural gas, hydrogen, welding, non-alloy steel, hardness, microstructure, heat input, pipe, X70

## Abstract

The expansion of the gas pipeline network makes it necessary, on the one hand, to meet the requirements of standards regarding the materials used, but on the other hand, it is necessary to weld them. In the case of natural gas as a fuel, the welding process is widely used, but in the case of replacing natural gas with a mixture of this gas and hydrogen, the requirements regarding the quality of the process must be significantly increased or the process must be completely changed. This article presents the results of testing welded joints for a newly developed welding technology for the transmission of a hydrogen mixture. Material tests were carried out on a butt-circumferential-welded joint made between two spiral pipes with an outer diameter of 711 mm and wall thickness of 11 mm in the X70 grade. The developed welding technology is distinguished by a change in the beveling method of the edges, which allows the heat input to the material to be limited. The technology was developed for use in natural on-shore and off-shore gas pipelines with the addition of hydrogen. As a result, additional requirements in terms of joint plasticity had to be met during welding. The test results obtained indicate that the joints are characterized by high strength (more than 581 MPa), higher than that of the base material (fracture in the base material) and good impact strength at reduced temperature (more than 129 J). In transverse corrosion, a hardness below 250 HV and a favorable structure of ferrite with different morphologies were obtained.

## 1. Introduction

In recent years, along with the growth of the global economy, there is a need to supply more energy, natural gas, and crude oil [1]. On the other hand, there is a tendency to use other energy sources, e.g., hydrogen, as they do not emit carbon dioxide into the atmosphere [2]. The construction of transmission networks, in particular gas pipelines, is a long-term process, and analyzing the individual stages from the concept to design and execution may take several years, while the construction of the entire gas pipeline network is an even longer process. This indicates that the materials used for gas pipelines must meet requirements not only in terms of the chemical composition of natural gas, but also other gaseous media that can ultimately be transported through such pipelines [3], affecting their corrosion conditions [4,5]. Hence, there are tendencies to change the approach in the field of materials used for pipelines, but also in the technology of their connection [6,7]. The dominant technology for connecting high-pressure gas pipelines is welding. This technology allows for the production of an infinitely long pipeline with preserved metallic continuity. However, there are many limitations resulting from the need to weld in the building yard, in conditions of variable temperature and humidity, and in forced positions [8,9]. As a result, with the thickness of the material and with the increase in the volume of the weld, the risk of welding defects or welding discontinuities increases, which may limit the possibility of pipeline operation in the future [8,9].

Among welding methods, an intensive development of arc welding has been observed, despite the fact that this technology has been used successfully for many years, but also of hybrid techniques [10,11]. Among the arc welding technologies, power sources based on surface tension transfer (STT), high-frequency pulse transfer, short circuit transfer, and other droplet transfer modes or assisted by a laser beam (laser–arc hybrid process) have been developed. In this case, arc welding is characterized by low efficiency, process control problems, especially for manual welding, and consequently nonuniform weld properties at the circumference of the pipe [12,13]. One of the solutions to these problems is the automation of the welding process and the possibility of using welding speeds much higher than those in the manual process. As a result of automation, an increase in the repeatability of welding production is observed, as well as an increase in efficiency with a reduced amount of heat input into the material [14,15,16,17,18,19,20]. The use of automated welding processes also makes it possible to change the method of preparing the edges for welding, as in manual welding the groove (bevel) must be wide so that the welder can reach the arc and observe the weld pool, and for the automated process it is unnecessary. This state of affairs made it possible to develop a technology for welding large-diameter pipes with a small wall thickness, where the V-bevel was changed to the U-bevel. The change in bevel also made it possible to make a welded joint with a reduced number of passes from 5–6 to 3, which significantly reduces the amount of heat input into the material. The reduction in the amount of heat input into the material is also influenced by a significant increase in welding speed (up to 2–3 m/min).

As a consequence of developing a new welding technology, it is also necessary to determine the achievable properties of the welded joints. Gas pipelines use non-alloy steels subjected to thermomechanical rolling or heat-treated high-strength steels, such as X65 (L420), X70 (L485), and X80 (L555), with a fine-grained ferritic or ferritic–bainite structure [21,22]. These steels are characterized by favorable mechanical and plastic properties, as well as good weldability. However, they require control of the amount of heat input, where failure to meet the conditions causes an increase in hardness in the heat-affected zone (HAZ) or grain growth. This parameter also affects the formation of the structure in the weld itself, where the appropriate amount of lamellar, fine-plate, or granular ferrite determines the impact strength of the joint.

The article presents the results of the test of a circumferential-welded joint in terms of meeting the requirements of the EN ISO 3183 [23] standard for gas pipelines. The test results obtained indicate that it is impossible to meet all the requirements due to the different mechanical properties of the strip from which spiral-welded pipes are made and the need to use welding wires dedicated to high-strength steel. Research shows that the newly developed solution is beneficial. Moreover, due to the necessity to adapt gas pipelines to the conditions of hydrogen transport in the area of the butt-welded joint, high impact strength is necessary, which in turn is a result of the structure created—a fine-grained ferrite-pearlite structure.

## 2. Materials and Methods

The test material was a butt-welded joint of spiral-welded pipes with a wall thickness of 11.0 mm. The nominal diameter of the pipe was 700 mm (outer diameter 711 mm). Spiral-welded pipes made of L485ME (X70) steel with the chemical composition and mechanical properties indicated in Table 1 were used for welding. The filler material used for welding was the electrode wire G55 4 M21 Mn3NiCrMo (OK AristoRod 55, ESAB, Vamberk, Czech Republic) according to EN ISO 16834-A [24] with a diameter of 1.2 mm. The chemical compositions of the filler metals according to the standard requirements are presented in Table 1. The used filler metal is dedicated to welding high-strength steel (with yield strength more than 550 MPa) and is not dedicated to welding high-pressure gas pipeline elements (based on the EN ISO 3183 [23] requirements). The chemical compositions included in the pipe documentation (according to EN 10204-3.1 [25]) were verified, allowing the actual chemical composition to be determined. The chemical composition analysis (“check”) of the base material and the weld was performed by optical emission spectroscopy (OES) using a Foundry-Master (WAS) spark spectrometer (Hitachi, Tokyo, Japan).

**Figure 1:**
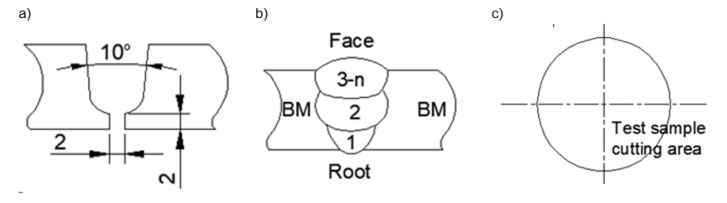
Welding conditions: (**a**) edge beveling; (**b**) seam sequence; and (**c**) sampling area for testing.

Circumferential welding was performed using the welding process 135 according to EN ISO 4063 [27] in a mechanized manner. The amount of heat input into the material was 0.84–0.85 kJ/mm. The shielding gas M21-ArC-10 according to EN ISO 14175 [28] (Ar + 10% CO_2_) was used. The preheat temperature was 100 °C and the interpass temperature was 150 °C. The preparation of the weld groove and the arrangement of the beads are shown in Figure 1. The welding parameters used are listed in Table 2.

After welding, the joint was subjected to non-destructive testing (NDT) to identify any welding imperfections. NDT tests included visual (VT), penetrant (PT), ultrasonic (UT-TOFD), and radiographic (RT) tests. The performed tests showed lack of welding imperfections and confirmed the proper shape of the weld. For the NDT test, the acceptance level was B (highest) according to EN ISO 5817 [29].

Macro- and microscopic examinations were carried out on metallographic cross sections after mechanical grinding and polishing. Water-abrasive papers were used for grinding, and Al_2_O_3_ water suspension was used for polishing. To reveal the microstructure, the samples were chemically etched in a 4% alcohol solution of nitric acid. The etching time was approximately 10 s. The microstructure was characterized using the light microscopes (LM) Leica Stereozoom S9i (Leica, Wetzlar, Germany) and Leica DM/LM (Leica, Wetzlar, Germany). Grain size percentages were assessed using the image analysis software Sigma Scan Pro (Version 5, 2007, Systat Software (version 5.0), San Jose, CA, USA) based on SEM images.

Hardness measurements were made using the Vickers method with an intender load of 10 kG (98.07 N), using a Zwick/Roell ZHU 187.5 hardness tester (Zwick Roell Group, Ulm, Germany).

The mechanical and plastic properties tests included static tensile, bending, and impact tests at +20 °C, 0 °C, −10 °C, −20 °C, and −30 °C. The static tensile test was performed on flat samples with a measuring length of 100 mm and a cross-section of 11.0 × 20 mm^2^. Impact tests were performed for the area of the base material (BM), HAZ, and weld (WM) using a standard sample 10 × 10 × 55 mm^3^ with a V-notch. After testing, the samples were subjected to fractographic observations using light microscopy (LM) and scanning electron microscopy—SEM (Phenom XL, Thermo Fisher Scientific, Waltham, MA, USA).

## 3. Results and Discussion

### 3.1. Chemical Composition

The chemical composition tests carried out indicate that the chemical composition of the base material and the weld (‘check’) was consistent with the chemical composition resulting from the smelting analysis (‘heat’) carried out in the smelter for the base material and indicated by the wire manufacturer (Table 1). The tests performed revealed that the joint material does not meet the chemical composition requirements for gas pipelines (EN ISO 3183 [23]) due to the increased content of Cr and Ni. Within this range, the permissible content of these elements is below 0.3%. The increased content of Cr and Ni is the result of the filler material used and is consistent with the requirements of the EN ISO 16834-A [24] standard. An increase in Cr (more than 0.5%) and Ni (more than 0.5%) content in relation to the base material occurs in the weld metal and is consistent with the chemical composition of the wire and is necessary to meet mechanical properties requirements.

### 3.2. Microscopic Examination

**Figure 2:**
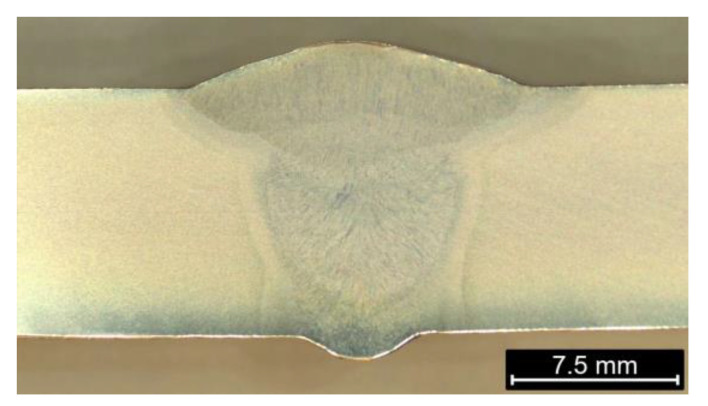
Macrostructure of butt weld of spiral-welded pipes.

The NDT tests did not reveal the occurrence of welding imperfections throughout the perimeter. For microscopic examination, four samples were taken every 90°. Figure 2 shows an example of a joint macrostructure. The joint was characterized by a small overflow of the face (up to 3 mm) and an outflow of the root (up to 1 mm). The joint was symmetrical with respect to the connected pipes. The width of the HAZ did not exceed 3 mm. Three seams were clearly visible and outlined, confirming the correctness of filling the weld groove. In the areas of the first and second seams, the visible areas changed due to the influence of heat input into the material.

**Figure 3:**
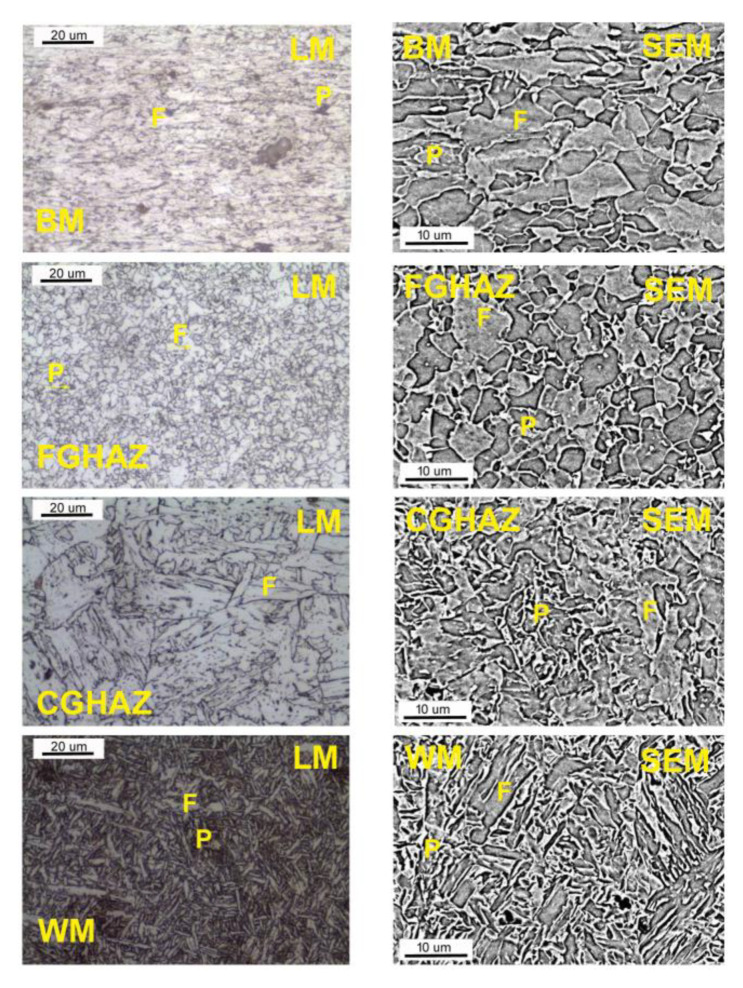
Microstructure of the butt-welded joint: BM—base metal, FGHAZ—fine-grain heat-affected zone, CGHAZ—coarse-grain heat-affected zone, WM—weld metal, F—ferrite, P—pearlite.

**Figure 4:**
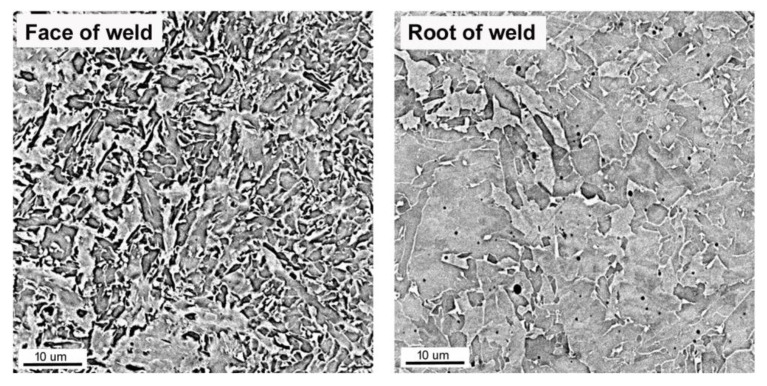
Microstructure of weld metal in the first (**left image**) and last seam (**right image**); SEM image.

Figure 3 shows the microstructure of individual areas of the welded joint observed using light microscopy (LM) and scanning electron microscopy (SEM). Microstructure studies in each area showed the presence of a fine-grained ferritic–pearlitic structure in the pipe material. The grain size analysis showed that the grain size was up to 10 μm. The areas of the perlite colonies were very small, and practically imperceptible. Due to the small percentage of pearlite in the structure, no clear structural changes were observed in the range of Ac1 and Ac3 temperatures, typical for unalloyed steels (Figure 3). In the HAZ area, two main areas were observed, i.e., the fine-grained area (FGHAZ) and the coarse-grained area (CGHAZ). Fragmentation of the structure was observed in the FGHAZ area. In the coarse-grained region, the presence of granular ferrite and Widmanstatten was observed (Figure 3). The facing bead in the weld had a fine-lamellar ferrite structure, typical of high-strength, high-impact steel welds (Figure 4). In the first and second passes (seam), the main structural component was fine plate ferrite. Apart from this, there was only a small amount (about 10%) of plate ferrite (Widmanstatten) and granular ferrite. In the root bead, the structure was similar except for the fact that larger amounts of lamellar and granular ferrite were observed (approx. 15–20%)—Figure 4.

By comparing the microstructures of the face and the root beads (Figure 4), it can be observed that heat input during subsequent seams caused structural changes. The occurrence of phase transformations indicates that the temperature in the root pass exceeds the Ac3 temperature. As a result of heating this area, the coarse crystalline structure resulting from the crystallization of the weld metal disappeared in the root area, but remained in the face area. The last seam was made in the face area and only cooled down after welding.

### 3.3. Mechanical Properties

The tests of mechanical properties were performed on samples from the strip and the joint. Samples covered the entire joint, i.e., base material (BM), heat-affected zone (HAZ), and weld (WM). The test revealed slightly lower mechanical properties than the strip material from which the pipe was made (strip). However, the yield strength (YS) and tensile strength (UTS) results were within the range required for butt-welded joints and met the requirements resulting from the pipe properties, that is above 485 MPa for yield strength and in the range of 570–760 MPa for tensile strength. Due to the use of a spiral-welded pipe in the tests, the strip material used was higher than the certificate results (Table 3). The obtained results, due to the anisotropy of the mechanical properties, were, respectively, 581 MPa (Joint 1) and 636 MPa (Joint 2) for the yield strength and 687 MPa and 693 MPa for the tensile strength. The total elongation of the welded joint was 18%. Fractographic studies performed with the use of SEM revealed the occurrence of a plastic fracture. The three-point bending test from the face and from the root side was successful, with a bend angle of 180° without cracks on the tensile surface (Table 3).

**Figure 5:**
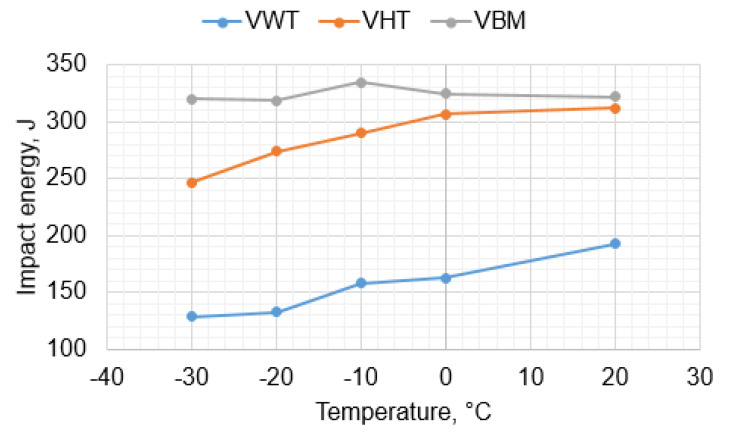
Impact energy curve for average values.

**Figure 6:**
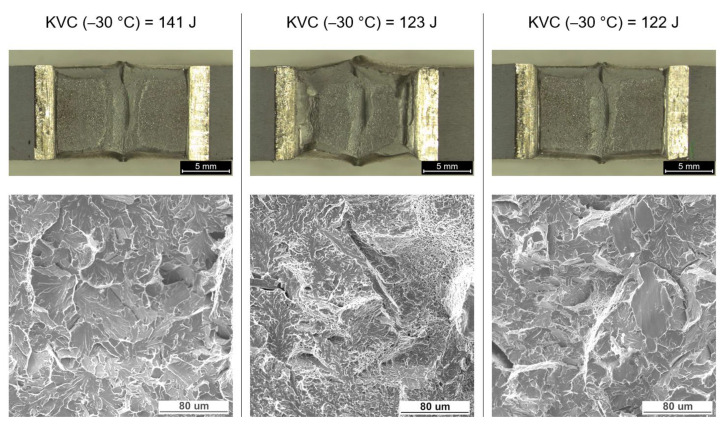
Fracture morphology for samples with the lowest impact strength of the weld material. Image using light microscopy (LM) and scanning electron microscopy (SEM).

Impact strength tests were carried out in the temperature range from +20 °C to −30 °C in the areas of BM, HAZ, and WM. The test results are presented in Table 4. The results obtained indicate the high plasticity of the material in each of the tested areas. For the base material, the impact energy was above 300 J, while in HAZ and WM a slight decrease in impact strength was observed to 193 J at +20 °C and to 129 J at −30 °C (Figure 5). At a temperature of −30 °C, a significant share of brittle fracture was also observed (Figure 6).

### 3.4. Hardness Testing

**Figure 7:**
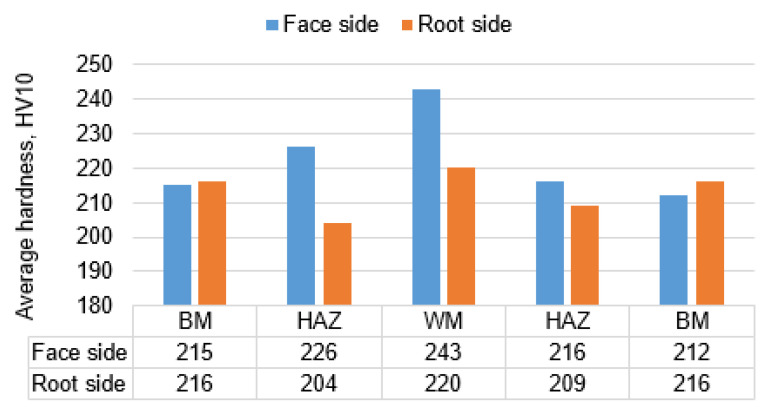
Average values of the hardness HV10 in specific areas of joint.

**Figure 8:**
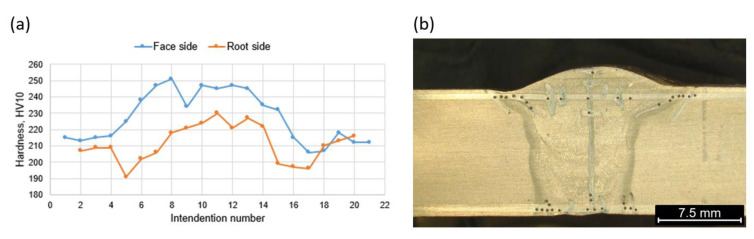
Hardness HV10 distribution in the joint cross section (**a**) and placement of intender (**b**).

The hardness measurements carried out in the cross-section showed that the welded joint was characterized by relatively low hardness. The lowest hardness was recorded in the base material, that is approximately 215 HV 10, while in HAZ and WM, an increase in hardness to 243 HV10 was observed in the area of the weld face (Figure 7). In the root area, the hardness was lower. In the HAZ, a hardness decrease was also observed, 204 HV10. A slight increase in hardness was observed in the weld area (220 HV10). Measurement results are shown in Figure 7 and Figure 8.

### 3.5. Discussion

The test of the butt-circumferential-welded joint using the mechanized method 135 showed that the consumables (filler metal) of the material group for welding high-strength steels are suitable to use in the construction of on-shore and off-shore high-pressure gas pipelines.

The results obtained show that the requirements regarding the chemical composition were not met, where as a result of the limited mixing of the base material with the metal of the wire, the total content of chromium and nickel was above the limit value. The chemical composition obtained from the weld was consistent with the chemical composition of the wire used. This indicates that the limited size of the weld groove and only three passes result in little mixing with the base material. The filler material used is dedicated to welding high-strength steel, which means that it is not compliant with the requirements of EN ISO 14341, but is appropriate for the mechanical and plastic properties of the strip used to produce the spirally welded pipe. The pipe itself, due to the coiling of the strip, is characterized by different properties in the longitudinal and circumferential directions, which is a consequence of the anisotropy of the mechanical properties resulting from rolling. The tests performed indicate that the mechanical properties (YS = 581 MPa) are higher than the strip certificate (YS = 629 MPa), but they are much higher than the requirements for the pipe (YS ≥ 485 MPa). The tensile strength is similar for the pipe and strip material (UTS = 687 MPa). The elongation obtained was slightly reduced from 22% to 18%, which is the effect of rolling the strip into a pipe, but the results obtained are also typical for testing the entire welded joint.

As a result of the different structures of WM and HAZ, these areas elongate differently from those of the base material. The results obtained are favorable for the operational reasons of the gas pipeline. The use of low welding linear energy (0.84 kJ/mm) caused a small amount of heat input, in the range of 0.45–0.91 kJ/mm. Low preheating (100 °C) and inter-pass (150 °C) temperatures allowed low hardness to be maintained in the area of the welded joint, where the highest values were observed in the area of the weld face (about 240 HV10). The hardness distribution was uniform and an increase in hardness was observed from approximately 215 HV10 for BM to 240 HV10 in the weld face area and 220 HV10 in the weld root. The relatively short cooling time, in turn, caused a lack of unfavorable martensitic–austenitic (M-A) islands in the joint structure. The absence of M-A islands ensured a high impact energy both at ambient temperature (more than 300 J for BM) and at reduced temperature (up to 129 J for WM). The decrease in impact strength in HAZ and WM was the result of a different structure in the welded joint relative to the base material. The base material has a fine ferrite structure with a grain size of less than 10 μm. In HAZ, it is fragmented into FGHAZ and slightly enlarged into CGHAZ. CGHAZ is dominated by Widmanstatten ferrite and granular ferrite, ensuring the high impact strength of this area (247 J at −30 °C). The weld is characterized by a slight inhomogeneity, which is the result of multi-pass welding. The root pass and filler beads were subjected to successive welding cycles, resulting in phase transitions. In the weld area, fine plate ferrite and a small amount of granular and Widmanstatten ferrite are observed. In the structure of the last run, the amount of granular ferrite and Widmanstatten was the smallest and their share was about 10%. Due to the properties of this structure, WM showed high impact strength (approximately 130 J at −30 °C), but also a fairly large share of brittle fractures.

## 4. Conclusions

The tests of the butt-welded joint of spirally welded steel pipes made of L485ME (X70) steel with the use of a filler material dedicated to welding high-strength steel yielded the following results:

- The requirements of EN ISO 3183 [23] regarding the chemical composition of the weld were not met. Slight excesses in Cr and Ni content were observed.
- The obtained welded joint was characterized by favorable mechanical properties higher than the pipe material. In the static tensile test, fracture occurred in the base material, and the tensile strength was close to the strength of the strip material from which the pipe was made (UTS = 688 MPa) and with high elongation (18%) and narrowing (more than 60%).
- The joint showed high impact strength throughout the operation range of high-pressure gas pipeline networks, where the lowest impact strength was recorded for WM and was 139 J at −30 °C. In other areas, the impact strength was higher, 247 J for HAZ and 320 J for BM, respectively. The largest share of brittle fracture was recorded for fractures with WM.
- As a result of the use of a bevel on the U, it was possible to reduce the amount of heat input to 0.83 kJ/mm and the number of stitches necessary to make it. The automated welding system enabled the use of high welding speeds, that is, 2.7 m/min for the root and 3.5 m/min for subsequent passes. The low amount of heat input ensured the favorable microstructure of the joint, while maintaining the high quality of the welded joints and proper penetration.
- The entire joint area was characterized by the presence of a ferritic structure. In BM and FGHAZ it was very fine, and in CGHAZ and WM the grains grew slightly. In these areas, in addition to fine plate ferrite, small amounts of plate and granular ferrite were also found.

## Figures and Tables

**Table 1:** Chemical composition of base metal and filler metal according to requirements and test results; % wag.

Document	C	Si	Mn	P	S	Cr	Ni	Mo	Cu	V	N	Other
ISO 3183 [23] (pipe)	≤0.12	≤0.45	≤1.7	≤0.025	≤0.015	≤0.30	≤0.50	≤0.35	≤0.50	≤0.11	≤0.012	
ISO 14341 [26] (wire)	0.06–0.14	0.4–1.3 *	0.9–1.9 *	≤0.025	≤0.025	≤0.15	≤0.15	≤0.15	≤0.35	≤0.03		
ISO 16834 [24] (wire)	≤0.14	**0.60–0.80**	**1.30–1.80**	≤0.015	≤0.018	**0.40–0.65**	**0.50–0.65**	0.15–0.30	≤0.30	≤0.030		≤0.25
Base metal (pipe)—heat	0.059	0.292	1.54	0.006	0.0007	0.22	0.026	0.113	0.018	0.002	0.0054	
Base metal (pipe)—check	0.054	0.302	1.54	0.006	0.001	0.221	0.025	0.116	0.019	0.002	0.0046	
EN 10204-3.1 [25] OK AristoRod 55	0.11	**0.77**	1.37	0.009	0.005	**0.53**	**0.53**	0.23	0.02	0.010		0.03
Weld metal—check	0.10	**0.62**	1.48	0.006	0.002	**0.49**	**0.43**	0.14	0.017	0.003		

*—extreme values from all included chemical compositions.

**Table 2:** Welding parameters.

Joint Area	Seam No	Voltage, V	Current, A	Welding Speed, mm/s	Wire Feed Speed, m/min	Heat Input, kJ/mm
Root	1	21	136	2700	6.4	0.85
Face	2-n	23	160	3500	6.8	0.84

**Table 3:** Static tensile test and bend test results.

Sample	Static Tensile Test				Bend Test	
	Yield Strength (YS), MPa	Tensile Strength (UTS), MPa	Elongation (Joint), %	Placement of Fracture	Former Diameter, mm	Bend Angle, °
Strip	629	688	22	-		
Pipe (required)	485	570–760	18			
Joint 1	581	687	19	BM	40	180
Joint 2	636	693	18	BM	40	180

**Table 4:** Impact energy test results: VWT—weld metal, VHT—heat-affected zone, VBM—base metal.

Joint Area	Test Temp., °C	KVC, J			Average
		1	2	3	
VWT	20	192	197	189	**193**
	0	161	159	169	**163**
	−10	154	162	158	**158**
	−20	131	128	139	**133**
	−30	141	123	122	**129**
VHT	20	313	318	304	**312**
	0	302	306	312	**307**
	−10	279	289	301	**290**
	−20	268	276	279	**274**
	−30	243	247	251	**247**
VBM	20	320	322	325	**322**
	0	312	314	348	**325**
	−10	343	329	334	**335**
	−20	307	329	321	**319**
	−30	329	330	300	**320**

## Data Availability

All data are provided in full in the Section 3 of this paper.

## References

[1] Rioux B., Shabaneh R., Griffiths S. (2021). An economic analysis of gas pipeline trade cooperation in the GCC. Energy Policy.

[2] Brown D., Reddi K., Elgowainy A. (2022). The development of natural gas and hydrogen pipeline capital cost estimating equations. Int. J. Hydrogen Energy.

[3] An T., Zheng S., Peng H., Wen X., Chen L., Zhang L. (2017). Synergistic action of hydrogen and stress concentration on the fatigue properties of X80 pipeline steel. Mater. Sci. Eng. A.

[4] Mustapha A., Charles E.A., Hardie D. (2012). Evaluation of environment-assisted cracking susceptibility of a grade X100 pipeline steel. Corros. Sci..

[5] Mousavi Anijdan S.H., Sabzi M., Park N., Lee U. (2022). Sour corrosion performance and sensitivity to hydrogen induced cracking in the X70 pipeline steel: Effect of microstructural variation and pearlite percentage. Int. J. Press. Vessel. Pip..

[6] Grossi Dornelas P.H., da Cruz Payao Filho J., Cipriano Farias F.W., Pereira Moraes e Oliveira V.H., de Oliveira Moraes D., Zumpano Júnior P. (2022). Influence of the interpass temperature on the microstructure and mechanical properties of the weld metal (AWS A5.18 ER70S-6) of a narrow gap welded API 5L X70 pipe joint. Int. J. Press. Vessel. Pip..

[7] Gu T., Zhang Q., Lian Z., Yu H., Chen J. (2021). Research and application of equivalent pipe model in stress analysis of lined pipe systems. Int. J. Press. Vessel. Pip..

[8] Zhu Z., Han J., Li H. (2015). Effect of alloy design on improving toughness for X70 steel during welding. Mater. Des..

[9] Hashemi S.H., Sedghi S., Soleymani V., Mohammadyani D. (2012). CTOA levels of welded joint in API X70 pipe steel. Eng. Fract. Mech..

[10] Sajek A. (2019). Application of FEM simulation method in area of the dynamics of cooling AHSS steel with a complex hybrid welding process. Weld. World.

[11] Kik T., Górka J. (2019). Numerical Simulations of Laser and Hybrid S700MC T-Joint Welding. Materials.

[12] Ghomashchi R., Costin W., Kurj R. (2015). Evolution of weld metal microstructure in shielded metal arc welding of X70 HSLA steel with cellulosic electrodes: A case study. Mater. Charact..

[13] Kryzhanivskyy Y., Poberezhny L., Maruschak P., Lyakh M., Slobodyan V., Zapukhliak V. (2019). Influence of test temperature on impact toughness of X70 pipe steel welds. Procedia Struct. Integr..

[14] Mousazadeh M.A., Derakhshandeh-Haghighi R. (2020). Autogenous tungsten inert gas welding of 430 ferritic stainless steel: The effect of interpass temperature on microstructure evolution and mechanical properties. J. Mater. Eng. Perform..

[15] Wang X.L., Tsai Y.T., Yang J.R., Wang Z.Q., Li X.C., Shang C.J., Misra R.D.K. (2017). Effect of interpass temperature on the microstructure and mechanical properties of multi-pass weld metal in a 550-MPa-grade offshore engineering steel. Weld. World.

[16] Jun J.H., Park J.H., Cheepu M., Cho S.M. (2019). Observation and analysis of metal transfer phenomena for high-current super-TIG welding process super-TIG welding process. Sci. Technol. Weld. Join..

[17] Martina F., Ding J., Williams S., Caballero A., Pardal G., Quintino L. (2019). Tandem metal inert gas process for high productivity wire arc additive manufacturing in stainless steel. Addit. Manuf..

[18] Nasilowska B., Slezak T., Sniezek L., Torzewski J. Mechanical Properties of Laser-Welded Joints in the Difficult-to-Weld Steels. Proceedings of the Intelligent Technologies in Logistics and Mechatronics Systems, ITELMS 2013—Proceedings of the 8th International Conference.

[19] Felipe L., Rossini S., André R., Reyes V., Spinelli J.E. (2019). Double-wire tandem GMAW welding process of HSLA50 steel. J. Manuf. Process..

[20] Tomków J., Świerczyńska A., Landowski M., Wolski A., Rogalski G. (2021). Bead-on-Plate Underwater Wet Welding on S700MC Steel. Adv. Sci. Technol. Res. J..

[21] Gordienko A.I., Derevyagina L.S., Malikov A.G., Orishich A.M., Surikova N.S., Volochaev M.N. (2020). The effect of the initial microstructure of the X70 low-carbon microalloyed steel on the heat affected zone formation and the mechanical properties of laser welded joints. Mater. Sci. Eng. A.

[22] Dalla V.K. (2022). Experimental investigation of fracture behavior and microstructure of API 5LX60 line pipe. Mater. Today Proc..

[23] (2019). Petroleum and Natural Gas Industries—Steel Pipe for Pipeline Transportation Systems.

[24] (2012). Welding Consumables—Wire Electrodes, Wires, Rods and Deposits for Gas Shielded Arc Welding of High Strength Steels—Classification.

[25] (2004). Metallic Products—Types of Inspection Documents.

[26] (2020). Welding Consumables—Wire Electrodes and Weld Deposits for Gas Shielded Metal Arc Welding of Non Alloy and Fine Grain Steels—Classification.

[27] (2011). Welding and Allied Processes—Nomenclature of Processes and Reference Numbers.

[28] (2008). Welding Consumables—Gases and Gas Mixtures for Fusion Welding and Allied Processes.

[29] (2023). Welding—Fusion-Welded Joints in Steel, Nickel, Titanium and Their Alloys (Beam Welding Excluded)—Quality Levels for Imperfections.

